# Tetra­aqua­(1,10-phenanthroline-κ^2^ *N*,*N*′)magnesium(II) bis­[(2,4-dichloro­phen­yl)acetate]

**DOI:** 10.1107/S1600536808022150

**Published:** 2008-07-23

**Authors:** Xiao-Min Hao, Chang-Sheng Gu, Weng-Dong Song, Ji-Wei Liu

**Affiliations:** a Department of Applied Chemistry, Guangdong Ocean University, Zhanjiang 524088, People’s Republic of China; b College of Chemistry and Chemical Technology, Daqing Petroleum Institute, Daqing 163318, People’s Republic of China

## Abstract

In the mononuclear title complex, [Mg(C_12_H_8_N_2_)(H_2_O)_4_](C_8_H_5_Cl_2_O_2_)_2_, each Mg^II^ ion is hexa­coordinated by two N atoms from a 1,10-phenanthroline ligand [Mg—N = 2.233 (2) Å] and four water mol­ecules [Mg—O*W* = 2.033 (2) and 2.043 (1) Å] in a distorted octa­hedral geometry. A twofold rotation axis passes through the Mg atom. In the crystal structure, the cations and anions are linked by inter­molecular O—H⋯O hydrogen bonds and π–π stacking inter­actions [centroid–centroid distance = 3.804 (2) Å] into layers parallel to the *ac* plane.

## Related literature

For related literature, see: Castellari *et al.* (1999 ▶); Kopylovich *et al.* (2003 ▶); Sharma *et al.* (2007 ▶); Zhou *et al.* (2007 ▶).
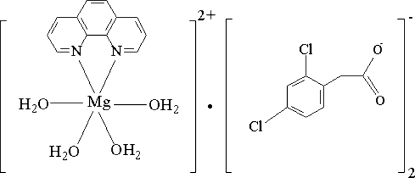


## Experimental

### 

#### Crystal data

[Mg(C_12_H_8_N_2_)(H_2_O)_4_](C_8_H_5_Cl_2_O_2_)_2_
*M* *_r_* = 684.62
Monoclinic, 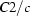

*a* = 28.926 (1) Å
*b* = 14.0447 (6) Å
*c* = 7.6074 (3) Å
β = 94.785 (1)°
*V* = 3079.8 (2) Å^3^
*Z* = 4
Mo *K*α radiation
μ = 0.46 mm^−1^
*T* = 273 (2) K
0.34 × 0.26 × 0.18 mm

#### Data collection

Bruker P4 diffractometer
Absorption correction: empirical **[OR multi-scan]**(using intensity measurements) (*SADABS*; Sheldrick, 1996 ▶) *T*_min_ = 0.867, *T*_max_ = 0.921
10955 measured reflections
3732 independent reflections
2619 reflections with *I* > 2σ(*I*)
*R*_int_ = 0.021

#### Refinement

*R*[*F* ^2^ > 2σ(*F* ^2^)] = 0.042
*wR*(*F* ^2^) = 0.117
*S* = 1.02
3732 reflections
207 parameters
6 restraints
H atoms treated by a mixture of independent and constrained refinement
Δρ_max_ = 0.36 e Å^−3^
Δρ_min_ = −0.47 e Å^−3^

### 

Data collection: *APEX2* (Bruker, 2004 ▶); cell refinement: *SAINT* (Bruker, 2004 ▶); data reduction: *SAINT*; program(s) used to solve structure: *SHELXS97* (Sheldrick, 2008 ▶); program(s) used to refine structure: *SHELXL97* (Sheldrick, 2008 ▶); molecular graphics: *SHELXTL* (Sheldrick, 2008 ▶); software used to prepare material for publication: *SHELXL97*.

## Supplementary Material

Crystal structure: contains datablocks I, global. DOI: 10.1107/S1600536808022150/im2068sup1.cif

Structure factors: contains datablocks I. DOI: 10.1107/S1600536808022150/im2068Isup2.hkl

Additional supplementary materials:  crystallographic information; 3D view; checkCIF report

## Figures and Tables

**Table 1:** Hydrogen-bond geometry (Å, °)

*D*—H⋯*A*	*D*—H	H⋯*A*	*D*⋯*A*	*D*—H⋯*A*
O2*W*—H2*W*1⋯O1^i^	0.849 (9)	1.876 (9)	2.725 (2)	178 (2)
O2*W*—H2*W*2⋯O1^ii^	0.841 (9)	1.96 (1)	2.772 (2)	161 (2)
O1*W*—H1*W*1⋯O2^iii^	0.851 (9)	1.91 (1)	2.728 (2)	162 (2)
O1*W*—H1*W*2⋯O2^i^	0.849 (9)	1.84 (1)	2.685 (2)	171 (2)

Symmetry codes: (i) 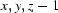; (ii) 
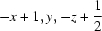
; (iii) 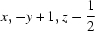.

**Fig. 1.**
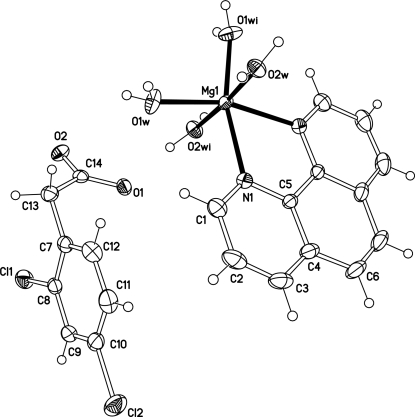
Molecular structure of (I) with 30% probability ellipsoids.

**Fig. 2.**
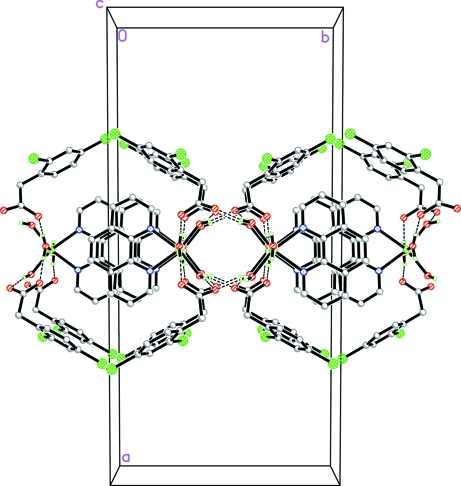
Packing diagram of (I).

## supplementary crystallographic information

### Comment

The rigid-type phenylacetic acid and its derivatives have versatile binding abilities and the potential capabilities of generating complicated supramolecular architectures. Nevertleless, main group or transition metal compounds in which the phenylacetate ion does not coordinate the metals are rare (Castellari *et al.* (1999), Kopylovich *et al.* (2003), Sharma *et al.* (2007), Zhou *et al.* (2007)). In the present study, we chose 2,4-dichlorophenylacetate as the anion to prepare a new mononuclear magnesium^II^ complex, [Mg(C_12_H_8_N_2_)(H_2_O)_4_][(C_8_H_5_Cl_2_O_2_)_2_], (I), the crystal structure of which is reported here.

As illustrated in Fig. 1, the asymmetric unit of (I) consists of one half of a [Mg(1,10-phen)(H_2_O)_4_] cation and one 2,4-dichlorophenylacetate anion. The Mg^II^ atom displays a distorted octahedral geometry defined by two N atoms from the 1,10-phenanthroline ligand [Mg—N 2.233 (2) Å] and four water molecules [Mg—Ow 2.033 (2), 2.043 (1) Å], respectively. The characteristic *C*—*O*(carboxylate) bond lengths suggest electron delocalization in the carboxylate groups of the anionic moieties. In the crystal structure, the cations and anions are linked by intermolecular O—H···O hydrogen bonds between the carboxylate O atoms and the coordinated water molecules. Additional π-π stacking interactions between 1,10-phen ligands with a distance of 3.804 (2) Å leads to the observation of layers parallel to the *ac*-plane (details see Table 1 and Fig. 2).

### Experimental

Benzyloxyacetic acid is commercially available and was used without further purification. The title complex was prepared by the addition of Mg(Cl)_2_ × 6 H_2_O (4.06 g, 20 mmol) and 1,10-phenanthroline (3.98 g, 20 mmol) to a hot aqueous solution of 2,4-dichlorophenylacetic acid (4.10 g, 20 mmol); the pH was adjusted to 6 with 0.1*M* sodium hydroxide. The solution was allowed to evaporate at room temperature. Colorless crystals separated from the filtered solution after several days. CHN analysis: Calcd. for C_28_H_26_N_2_O_8_Cl_4_Mg: C 49.12, H 3.83, N 4.09%. Found: C 49.14, H 3.82, 4.08%.

### Refinement

The H atoms attached to C atoms were placed in calculated positions, with C—H = 0.93 Å and *U*_iso_(H) = 1.2*U*_eq_(C). The H atoms of water molecule were located in a difference Fourier map and refined with O—H distance restraint of 0.85 (1) Å, and *U*_iso_(H) = 1.5*U*_eq_(O).

### Crystal data

**Table:**

[Mg(C_12_H_8_N_2_)(H_2_O)_4_](C_8_H_5_Cl_2_O_2_)_2_	*F*_000_ = 1408
*M**_r_* = 684.62	*D*_x_ = 1.477 Mg m^−^^3^
Monoclinic, *C*2/*c*	Mo *K*α radiation λ = 0.71073 Å
Hall symbol: -C 2yc	Cell parameters from 10366 reflections
*a* = 28.926 (1) Å	θ = 2.8–28.2º
*b* = 14.0447 (6) Å	µ = 0.46 mm^−^^1^
*c* = 7.6074 (3) Å	*T* = 273 (2) K
β = 94.785 (1)º	Prism, colorless
*V* = 3079.8 (2) Å^3^	0.34 × 0.26 × 0.18 mm
*Z* = 4	

### Data collection

**Table:**

Bruker P4 diffractometer	3732 independent reflections
Radiation source: fine-focus sealed tube	2619 reflections with *I* > 2σ(*I*)
Monochromator: graphite	*R*_int_ = 0.021
Detector resolution: 10.000 pixels mm^-1^	θ_max_ = 28.2º
*T* = 273(2) K	θ_min_ = 2.8º
ω scans	*h* = −38→33
Absorption correction: empirical (using intensity measurements)(SADABS; Sheldrick, 1996)	*k* = −18→18
*T*_min_ = 0.867, *T*_max_ = 0.921	*l* = −9→10
10955 measured reflections	

### Refinement

**Table:**

Refinement on *F*^2^	Secondary atom site location: difference Fourier map
Least-squares matrix: full	Hydrogen site location: inferred from neighbouring sites
*R*[*F*^2^ > 2σ(*F*^2^)] = 0.042	H atoms treated by a mixture of independent and constrained refinement
*wR*(*F*^2^) = 0.118	*w* = 1/[σ^2^(*F*_o_^2^) + (0.0514*P*)^2^ + 1.7903*P*] where *P* = (*F*_o_^2^ + 2*F*_c_^2^)/3
*S* = 1.03	(Δ/σ)_max_ < 0.001
3732 reflections	Δρ_max_ = 0.36 e Å^−^^3^
207 parameters	Δρ_min_ = −0.47 e Å^−^^3^
6 restraints	Extinction correction: SHELXL97 (Sheldrick, 2008)
Primary atom site location: structure-invariant direct methods	Extinction coefficient: 0.0001 (1)

### Fractional atomic coordinates and isotropic or equivalent isotropic displacement parameters (Å^2^)

**Table:**

	*x*	*y*	*z*	*U*_iso_*/*U*_eq_	
Mg1	0.5000	0.70119 (6)	0.2500	0.0377 (2)	
Cl1	0.30958 (2)	0.68124 (5)	0.95908 (8)	0.06695 (19)	
Cl2	0.25982 (3)	0.96821 (5)	0.52259 (13)	0.0957 (3)	
O1	0.42857 (5)	0.68303 (10)	0.76580 (19)	0.0501 (3)	
O1W	0.44481 (6)	0.61206 (10)	0.20734 (19)	0.0613 (4)	
O2W	0.50764 (5)	0.69659 (11)	−0.01445 (17)	0.0480 (3)	
O2	0.41678 (5)	0.54277 (10)	0.88709 (19)	0.0544 (4)	
N1	0.45496 (5)	0.82808 (11)	0.1896 (2)	0.0423 (4)	
C1	0.41135 (7)	0.82785 (17)	0.1205 (3)	0.0577 (6)	
H1A	0.3966	0.7697	0.0991	0.069*	
C2	0.38664 (9)	0.9115 (2)	0.0787 (4)	0.0733 (7)	
H2A	0.3562	0.9084	0.0293	0.088*	
C3	0.40715 (9)	0.9969 (2)	0.1101 (3)	0.0705 (7)	
H3A	0.3907	1.0527	0.0846	0.085*	
C4	0.45322 (8)	1.00081 (15)	0.1812 (3)	0.0552 (5)	
C5	0.47614 (6)	0.91362 (12)	0.2161 (2)	0.0392 (4)	
C6	0.47783 (10)	1.08762 (15)	0.2176 (4)	0.0734 (8)	
H6A	0.4627	1.1453	0.1957	0.088*	
C7	0.33544 (7)	0.68867 (14)	0.6254 (3)	0.0457 (5)	
C8	0.31108 (6)	0.73310 (14)	0.7514 (3)	0.0456 (4)	
C9	0.28790 (7)	0.81845 (15)	0.7222 (3)	0.0544 (5)	
H9A	0.2720	0.8466	0.8101	0.065*	
C10	0.28898 (7)	0.86044 (16)	0.5596 (3)	0.0603 (6)	
C11	0.31228 (9)	0.81981 (19)	0.4301 (3)	0.0698 (7)	
H11A	0.3128	0.8491	0.3206	0.084*	
C12	0.33515 (8)	0.73431 (19)	0.4637 (3)	0.0628 (6)	
H12A	0.3508	0.7066	0.3750	0.075*	
C13	0.36083 (7)	0.59691 (15)	0.6623 (3)	0.0532 (5)	
H13A	0.3682	0.5690	0.5514	0.064*	
H13B	0.3406	0.5530	0.7176	0.064*	
C14	0.40558 (7)	0.60887 (14)	0.7815 (2)	0.0413 (4)	
H2W1	0.4831 (4)	0.6910 (16)	−0.083 (2)	0.062*	
H2W2	0.5312 (4)	0.6889 (16)	−0.071 (2)	0.062*	
H1W1	0.4397 (8)	0.5689 (12)	0.2818 (19)	0.062*	
H1W2	0.4390 (8)	0.5895 (14)	0.1044 (13)	0.062*	

### Atomic displacement parameters (Å^2^)

**Table:**

	*U*^11^	*U*^22^	*U*^33^	*U*^12^	*U*^13^	*U*^23^
Mg1	0.0472 (5)	0.0291 (4)	0.0373 (4)	0.000	0.0060 (4)	0.000
Cl1	0.0679 (4)	0.0739 (4)	0.0613 (4)	0.0017 (3)	0.0193 (3)	0.0105 (3)
Cl2	0.0834 (5)	0.0587 (4)	0.1391 (7)	0.0047 (3)	−0.0259 (5)	0.0226 (4)
O1	0.0404 (7)	0.0532 (8)	0.0568 (9)	−0.0033 (6)	0.0054 (6)	0.0051 (6)
O1W	0.0958 (12)	0.0482 (8)	0.0394 (8)	−0.0322 (8)	0.0021 (8)	0.0015 (6)
O2W	0.0441 (8)	0.0631 (9)	0.0373 (7)	0.0031 (7)	0.0062 (6)	0.0027 (6)
O2	0.0743 (10)	0.0418 (7)	0.0463 (8)	0.0029 (7)	−0.0003 (7)	−0.0041 (6)
N1	0.0421 (9)	0.0402 (8)	0.0452 (9)	0.0014 (7)	0.0074 (7)	0.0005 (7)
C1	0.0458 (12)	0.0650 (14)	0.0623 (14)	0.0013 (10)	0.0035 (10)	0.0007 (11)
C2	0.0489 (13)	0.095 (2)	0.0760 (17)	0.0240 (14)	0.0029 (11)	0.0105 (15)
C3	0.0744 (17)	0.0695 (16)	0.0687 (16)	0.0356 (14)	0.0129 (13)	0.0151 (13)
C4	0.0762 (15)	0.0408 (10)	0.0513 (12)	0.0170 (10)	0.0204 (11)	0.0065 (9)
C5	0.0504 (10)	0.0320 (8)	0.0368 (9)	0.0040 (8)	0.0137 (7)	0.0022 (7)
C6	0.114 (2)	0.0323 (10)	0.0777 (18)	0.0144 (11)	0.0287 (16)	0.0067 (11)
C7	0.0366 (10)	0.0509 (11)	0.0486 (11)	−0.0043 (8)	−0.0024 (8)	−0.0053 (9)
C8	0.0377 (10)	0.0482 (11)	0.0509 (11)	−0.0053 (8)	0.0037 (8)	0.0014 (9)
C9	0.0407 (11)	0.0514 (12)	0.0710 (15)	−0.0013 (9)	0.0041 (10)	−0.0032 (10)
C10	0.0440 (12)	0.0514 (12)	0.0827 (17)	−0.0039 (10)	−0.0108 (11)	0.0088 (12)
C11	0.0623 (15)	0.0838 (18)	0.0612 (15)	−0.0099 (13)	−0.0061 (12)	0.0236 (13)
C12	0.0558 (13)	0.0827 (17)	0.0498 (12)	−0.0009 (12)	0.0034 (10)	−0.0016 (12)
C13	0.0486 (12)	0.0500 (12)	0.0602 (13)	0.0000 (9)	−0.0006 (9)	−0.0155 (10)
C14	0.0430 (10)	0.0424 (10)	0.0395 (10)	0.0058 (8)	0.0095 (8)	−0.0086 (8)

### Geometric parameters (Å, °)

**Table:**

Mg1—N1	2.2327 (16)	C3—C4	1.397 (3)
Mg1—O1W	2.0333 (15)	C3—H3A	0.9300
Mg1—O2W	2.0432 (13)	C4—C5	1.407 (3)
Mg1—N1^i^	2.2327 (16)	C4—C6	1.427 (3)
Mg1—O1W^i^	2.0333 (15)	C5—C5^i^	1.433 (4)
Mg1—O2W^i^	2.0432 (13)	C6—C6^i^	1.335 (6)
Cl1—C8	1.744 (2)	C6—H6A	0.9300
Cl2—C10	1.744 (2)	C7—C8	1.385 (3)
N1—C1	1.325 (3)	C7—C12	1.386 (3)
N1—C5	1.356 (2)	C7—C13	1.498 (3)
O1—C14	1.247 (2)	C8—C9	1.382 (3)
O2—C14	1.252 (2)	C9—C10	1.373 (3)
O1W—H1W1	0.851 (9)	C9—H9A	0.9300
O1W—H1W2	0.849 (9)	C10—C11	1.365 (4)
O2W—H2W1	0.849 (9)	C11—C12	1.384 (3)
O2W—H2W2	0.841 (9)	C11—H11A	0.9300
C1—C2	1.399 (3)	C12—H12A	0.9300
C1—H1A	0.9300	C13—C14	1.527 (3)
C2—C3	1.350 (4)	C13—H13A	0.9700
C2—H2A	0.9300	C13—H13B	0.9700
			
N1^i^—Mg1—N1	74.07 (8)	C3—C4—C6	123.6 (2)
O1W—Mg1—N1	91.24 (6)	C4—C3—H3A	120.2
O1W^i^—Mg1—N1	163.97 (7)	C4—C5—C5^i^	119.50 (13)
O1W—Mg1—O1W^i^	104.01 (11)	C4—C6—H6A	119.3
O1W—Mg1—O2W	88.36 (6)	C5—N1—Mg1	115.34 (12)
O1W^i^—Mg1—O2W	89.41 (6)	C5—C4—C6	119.2 (2)
O2W^i^—Mg1—N1	96.82 (6)	C6^i^—C6—C4	121.32 (14)
O2W—Mg1—N1	86.08 (6)	C6^i^—C6—H6A	119.3
O2W^i^—Mg1—O2W	176.38 (9)	C7—C8—Cl1	119.45 (15)
Mg1—O1W—H1W1	120.6 (15)	C7—C12—H12A	118.9
Mg1—O1W—H1W2	118.1 (15)	C7—C13—C14	113.25 (16)
Mg1—O2W—H2W1	117.2 (14)	C7—C13—H13A	108.9
Mg1—O2W—H2W2	131.6 (14)	C7—C13—H13B	108.9
N1—C1—C2	122.7 (2)	C8—C7—C12	116.1 (2)
N1—C1—H1A	118.7	C8—C7—C13	121.84 (19)
N1—C5—C4	122.90 (18)	C8—C9—H9A	121.0
N1—C5—C5^i^	117.60 (10)	C9—C8—C7	123.2 (2)
O1—C14—O2	124.69 (18)	C9—C8—Cl1	117.34 (17)
O1—C14—C13	117.88 (18)	C9—C10—Cl2	118.1 (2)
O2—C14—C13	117.42 (18)	C10—C9—C8	118.0 (2)
O1W—Mg1—N1^i^	163.97 (7)	C10—C9—H9A	121.0
O1W^i^—Mg1—N1^i^	91.24 (6)	C10—C11—C12	119.1 (2)
O1W—Mg1—O2W^i^	89.41 (6)	C10—C11—H11A	120.5
O1W^i^—Mg1—O2W^i^	88.36 (6)	C11—C10—C9	121.4 (2)
O2W^i^—Mg1—N1^i^	86.08 (6)	C11—C10—Cl2	120.5 (2)
O2W—Mg1—N1^i^	96.82 (6)	C11—C12—C7	122.2 (2)
C1—N1—C5	117.67 (17)	C11—C12—H12A	118.9
C1—N1—Mg1	126.82 (14)	C12—C7—C13	122.1 (2)
C1—C2—H2A	120.1	C12—C11—H11A	120.5
C2—C1—H1A	118.7	C14—C13—H13A	108.9
C2—C3—C4	119.7 (2)	C14—C13—H13B	108.9
C2—C3—H3A	120.2	H1W1—O1W—H1W2	108.5 (14)
C3—C2—C1	119.8 (2)	H2W1—O2W—H2W2	110.2 (14)
C3—C2—H2A	120.1	H13A—C13—H13B	107.7
C3—C4—C5	117.2 (2)		
			
Mg1—N1—C1—C2	−176.53 (18)	C5—N1—C1—C2	−1.5 (3)
Mg1—N1—C5—C4	178.64 (15)	C5—C4—C6—C6^i^	0.6 (5)
Mg1—N1—C5—C5^i^	−1.9 (3)	C6—C4—C5—N1	178.3 (2)
N1^i^—Mg1—N1—C1	175.8 (2)	C6—C4—C5—C5^i^	−1.2 (3)
N1^i^—Mg1—N1—C5	0.66 (9)	C7—C8—C9—C10	0.5 (3)
N1—C1—C2—C3	−0.6 (4)	C7—C13—C14—O1	−35.1 (3)
O1W—Mg1—N1—C1	−10.68 (18)	C7—C13—C14—O2	145.54 (19)
O1W^i^—Mg1—N1—C1	151.6 (2)	C8—C7—C12—C11	0.6 (3)
O1W—Mg1—N1—C5	174.14 (13)	C8—C7—C13—C14	−74.1 (2)
O1W^i^—Mg1—N1—C5	−23.6 (3)	C8—C9—C10—C11	−0.2 (3)
O2W^i^—Mg1—N1—C1	−100.24 (18)	C8—C9—C10—Cl2	−179.92 (15)
O2W—Mg1—N1—C1	77.59 (18)	C9—C10—C11—C12	0.2 (4)
O2W^i^—Mg1—N1—C5	84.59 (13)	C10—C11—C12—C7	−0.4 (4)
O2W—Mg1—N1—C5	−97.59 (13)	Cl1—C8—C9—C10	179.64 (16)
C1—N1—C5—C4	3.0 (3)	C12—C7—C8—C9	−0.7 (3)
C1—N1—C5—C5^i^	−177.5 (2)	Cl2—C10—C11—C12	179.89 (17)
C1—C2—C3—C4	1.2 (4)	C13—C7—C8—C9	179.01 (18)
C2—C3—C4—C5	0.2 (4)	C12—C7—C8—Cl1	−179.82 (15)
C2—C3—C4—C6	179.5 (2)	C12—C7—C13—C14	105.5 (2)
C3—C4—C5—N1	−2.4 (3)	C13—C7—C8—Cl1	−0.2 (3)
C3—C4—C6—C6^i^	−178.7 (3)	C13—C7—C12—C11	−179.0 (2)
C3—C4—C5—C5^i^	178.1 (2)		

Symmetry codes: (i) −*x*+1, *y*, −*z*+1/2.

### Hydrogen-bond geometry (Å, °)

**Table:**

*D*—H···*A*	*D*—H	H···*A*	*D*···*A*	*D*—H···*A*
O2W—H2W1···O1^ii^	0.849 (9)	1.876 (9)	2.725 (2)	178 (2)
O2W—H2W2···O1^i^	0.841 (9)	1.96 (1)	2.772 (2)	161 (2)
O1W—H1W1···O2^iii^	0.851 (9)	1.91 (1)	2.728 (2)	162 (2)
O1W—H1W2···O2^ii^	0.849 (9)	1.84 (1)	2.685 (2)	171 (2)

Symmetry codes: (ii) *x*, *y*, *z*−1; (i) −*x*+1, *y*, −*z*+1/2; (iii) *x*, −*y*+1, *z*−1/2.
